# Supplementary material for: Co-option of neck muscles supported the vertebrate water-to-land transition
Source: Nat Commun. 2024 Dec 4;15:10564. doi: 10.1038/s41467-024-54724-x (PMC11618326; doi:10.1038/s41467-024-54724-x)
Supplement: Supplementary file 3 — Description of Additional Supplementary Files [file 41467_2024_54724_MOESM3_ESM.pdf]

Description of Additional Supplementary Files

File Name: Supplementary Movie 1
Description: 3D interactive view of the neuromuscular system at the head/trunk transition of a 5 dpf zebrafish larva to complete data shown in Fig. 2j-l and Supplementary Fig. 2a-d.

File Name: Supplementary Movie 2
Description: 3D interactive view of the neuromuscular system at the head/trunk transition of a 7 dpf zebrafish larva to complete data shown in Supplementary Fig. 2e-o.

File Name: Supplementary Movie 3
Description: 3D interactive view of the neuromuscular system at the head/trunk transition of an axolotl larva to complete data shown in Supplementary Fig. 3.
