# Supplementary material for: Co-option of neck muscles supported the vertebrate water-to-land transition
Source: Nat Commun. 2024 Dec 4;15:10564. doi: 10.1038/s41467-024-54724-x (PMC11618326; doi:10.1038/s41467-024-54724-x)
Supplement: Supplementary file 7 — Reporting Summary [file 41467_2024_54724_MOESM7_ESM.pdf]

nature portfolio

Corresponding author(s): HEUDE Eglantine

Last updated by author(s): Nov 6, 2024

Reporting Summary

Nature Portfolio wishes to improve the reproducibility of the work that we publish. This form provides structure for consistency and transparency in reporting. For further information on Nature Portfolio policies, see our Editorial Policies and the Editorial Policy Checklist.

Statistics

For all statistical analyses, confirm that the following items are present in the figure legend, table legend, main text, or Methods section.

n/a	Confirmed	
☐	☒	The exact sample size (*n*) for each experimental group/condition, given as a discrete number and unit of measurement
☒	☐	A statement on whether measurements were taken from distinct samples or whether the same sample was measured repeatedly
☒	☐	The statistical test(s) used AND whether they are one- or two-sided<br>*Only common tests should be described solely by name; describe more complex techniques in the Methods section.*
☒	☐	A description of all covariates tested
☒	☐	A description of any assumptions or corrections, such as tests of normality and adjustment for multiple comparisons
☒	☐	A full description of the statistical parameters including central tendency (e.g. means) or other basic estimates (e.g. regression coefficient) AND variation (e.g. standard deviation) or associated estimates of uncertainty (e.g. confidence intervals)
☒	☐	For null hypothesis testing, the test statistic (e.g. *F*, *t*, *r*) with confidence intervals, effect sizes, degrees of freedom and *P* value noted<br>*Give P values as exact values whenever suitable.*
☒	☐	For Bayesian analysis, information on the choice of priors and Markov chain Monte Carlo settings
☒	☐	For hierarchical and complex designs, identification of the appropriate level for tests and full reporting of outcomes
☒	☐	Estimates of effect sizes (e.g. Cohen's *d*, Pearson's *r*), indicating how they were calculated

Our web collection on statistics for biologists contains articles on many of the points above.

Software and code

Policy information about availability of computer code

Data collection	Zen Software (Carl Zeiss, Germany)
Data analysis	Zen and Arivis Software (Carl Zeiss, Germany) / Mimics Innovation Suite 19 software (Materialise SV) / Blender (Blender Foundation)

For manuscripts utilizing custom algorithms or software that are central to the research but not yet described in published literature, software must be made available to editors and reviewers. We strongly encourage code deposition in a community repository (e.g. GitHub). See the Nature Portfolio guidelines for submitting code & software for further information.

Data

Policy information about availability of data

All manuscripts must include a data availability statement. This statement should provide the following information, where applicable:

- Accession codes, unique identifiers, or web links for publicly available datasets
- A description of any restrictions on data availability
- For clinical datasets or third party data, please ensure that the statement adheres to our policy

All data supporting the findings of this study are available within the paper and its Supplementary Information.

Research involving human participants, their data, or biological material

Policy information about studies with human participants or human data. See also policy information about sex, gender (identity/presentation), and sexual orientation and race, ethnicity and racism.

Reporting on sex and gender	N/A
Reporting on race, ethnicity, or other socially relevant groupings	N/A
Population characteristics	N/A
Recruitment	N/A
Ethics oversight	N/A

Note that full information on the approval of the study protocol must also be provided in the manuscript.

Field-specific reporting

Please select the one below that is the best fit for your research. If you are not sure, read the appropriate sections before making your selection.

☒ Life sciences ☐ Behavioural & social sciences ☐ Ecological, evolutionary & environmental sciences

For a reference copy of the document with all sections, see nature.com/documents/nr-reporting-summary-flat.pdf

Life sciences study design

All studies must disclose on these points even when the disclosure is negative.

Sample size	In experiments involving zebrafish embryos, no statistical methods were used to pre-determine sample sizes but our sample sizes are similar to those reported in previous publications [Nature 618, 543–549 (2023); Nature Comm 15, 6313 (2024)].
Data exclusions	Embryos with weak GFP fluorescence were excluded from the analysis
Replication	Experimental procedures has been performed at least in triplicates and we confirm that all experimental conditions and findings showed consistency (see for instance Supp. Fig. 1)
Randomization	All samples have been randomized in experimental procedures
Blinding	Blinding was not relevant as group allocation for data collection and analysis was not suitable in our study

Reporting for specific materials, systems and methods

We require information from authors about some types of materials, experimental systems and methods used in many studies. Here, indicate whether each material, system or method listed is relevant to your study. If you are not sure if a list item applies to your research, read the appropriate section before selecting a response.

Materials & experimental systems

n/a	Involved in the study	
☐	☒	Antibodies
☒	☐	Eukaryotic cell lines
☒	☐	Palaeontology and archaeology
☐	☒	Animals and other organisms
☒	☐	Clinical data
☒	☐	Dual use research of concern
☒	☐	Plants

Methods

n/a	Involved in the study	
☒	☐	ChIP-seq
☒	☐	Flow cytometry
☒	☐	MRI-based neuroimaging

Antibodies

Antibodies used	- anti-green fluorescent protein (GFP), Abcam, ab13970, dilution 1/1000<br>- anti-Myosin Heavy Chain (MyHC) DHSB clone A4.1025, dilution 1/20<br>- anti-acetylated tubulin (ac-Tub) Sigma-Aldrich T7451, 1/500

- goat anti-chick Alexa 488 (Invitrogen A-11039), dilution 1/500
- goat anti-mouse Alexa 555-IgG2a (Invitrogen A-21137), dilution 1/500
- goat anti-mouse Alexa 647-IgG2b (Invitrogen A-21242), dilution 1/500

All antibodies used in the context of the study were previously validated in the species and in the literature in an extensive manner

Validation

All validation and relevant references are reported in the supplier websites (see below the link for each antibody)
- anti-GFP : https://www.abcam.com/en-fr/products/primary-antibodies/gfp-antibody-ab13970#
- anti-MyHC : https://dshb.biology.uiowa.edu/A4-1025
- anti-ac-Tub : https://www.sigmaaldrich.com/FR/fr/product/sigma/t7451
- goat anti-chick Alexa 488 : https://www.thermofisher.com/antibody/product/Goat-anti-Chicken-IgY-H-L-Secondary-Antibody-Polyclonal/A-11039
- goat anti-mouse Alexa 555-IgG2a : https://www.thermofisher.com/antibody/product/Goat-anti-Mouse-IgG2a-Cross-Adsorbed-Secondary-Antibody-Polyclonal/A-21137
- goat anti-mouse Alexa 647-IgG2b : https://www.thermofisher.com/antibody/product/Goat-anti-Mouse-IgG2b-Cross-Adsorbed-Secondary-Antibody-Polyclonal/A-21242

Animals and other research organisms

Policy information about studies involving animals; ARRIVE guidelines recommended for reporting animal research, and Sex and Gender in Research

Laboratory animals

- Danio rerio: WT strain (*AB), Tg(Pax3a:eGFP), Tg(-1.5hsp70l:loxP-STOP-loxP-EGFP, cryaa:Venus), Tg(-3.2tbx1:creERT2;cryaa:Venus) at embryonic stage 1-2-3-5-7 dpf and adults
- Ambystoma mexicanum: Albino (tyr) at embryonic stage 46, one juvenile and adults

Wild animals

All wild animals (bichir, coelacanth, lungfish, newt and lizard) were fluid-preserved natural science specimens from laboratory and museum collections

Reporting on sex

Sex and gender aspects were not relevant to the biological process analyzed in larvae and juvenile specimens included in the study

Field-collected samples

No sample has been collected in the field in the context of the study

Ethics oversight

Laboratory animals were maintained in agreement with recommendations of the national authorities of Switzerland (Animal Protection Ordinance) and following the regulation of the 2010/63 UE European directives. Protocols were approved by the cantonal veterinary office of the Canton Zurich (Kantonales Veterinäramt, permit no. 150) and by the veterinary office of the IACUC of the University of Colorado School of Medicine (protocol no. 00979), Aurora, Colorado.

Note that full information on the approval of the study protocol must also be provided in the manuscript.

Plants

Seed stocks

N/A

Novel plant genotypes

N/A

Authentication

N/A
